# SOX2OT knockdown derived changes in mitotic regulatory gene network of cancer cells

**DOI:** 10.1186/s12935-018-0618-8

**Published:** 2018-09-05

**Authors:** Marie Saghaeian Jazi, Nader Mansour Samaei, Seyed Javad Mowla, Babak Arefnezhad, Morteza Kouhsar

**Affiliations:** 1 Metabolic Disorders Research Center, Golestan University of Medical Sciences, Gorgan, Iran; 2 Stem Cell Research Center, Golestan University of Medical Sciences, Po Box: 4934174611, Gorgan, Iran; 3 Department of Molecular Genetics, Faculty of Biological Sciences, Tarbiat Modares University, Tehran, Iran; 4 OMICS™ Research Group (Media Teb Gene), Tehran, Iran; 5 Laboratory of System Biology and Bioinformatics (LBB), University of Tehran, Institute of Biochemistry and Biophysics, Tehran, Iran

**Keywords:** *SOX2* overlapping transcript, Cell cycle, Cancer cell

## Abstract

**Background:**

SOX2 overlapping transcript (SOX2OT) is a long non-coding RNA, over-expressed in human tumor tissues and embryonic cells. Evidences support its function in the cell cycle; however there is no clear mechanism explaining its function in cell proliferation regulation. Here we investigated cancer cell response to SOX2OT knockdown by RNA sequencing.

**Methods:**

SOX2OT expression was inhibited by siRNA in two cancer cell lines (A549, U-87 MG), then the RNA of treated cells were used for the cDNA library synthesis and RNA sequencing. The differentially expressed genes were used for functional enrichment and the gene expression network was analyzed to find the most relevant biological process with SOX2OT function. Furthermore, the expression change of candidate genes was measured by qRT-PCR for more confirmation and the cell cycle was monitored by PI staining.

**Results:**

Our findings showed that SOX2OT knockdown affects the cellular gene expression generally with enriched cell proliferation and development biological process. Particularly, the cell cycle and mitotic regulatory genes expression including: *CDK2*, *CDK2AP2*, *ACTR3*, and chromosome structure associated genes like *SMC4*,* INCENP* and* GNL3L* are changed in treated cancer cells.

**Conclusion:**

Our results propound SOX2OT association with cell cycle and mitosis regulation in cancer cells.

**Electronic supplementary material:**

The online version of this article (10.1186/s12935-018-0618-8) contains supplementary material, which is available to authorized users.

## Background

Long non-coding RNAs (lncRNAs) are mRNA like ribonucleic acids with no protein products. Generally, they act in a wide range of cellular and molecular processes including chromatin remodeling [1–3], gene regulation [4, 5], proliferation [6, 7], metastasis [8–10] and etc. As respect to their key functions; there are many lncRNAs reported to be associated with human diseases [11–13].

*SOX2OT* is a lncRNA located in chr3q:26which overlaps *SOX2* gene in sequence [14, 15]. The *SOX2OT* expression is de-regulated in human cancer tissues [16–18] and its expression decrease during differentiation of cells [14, 18]. Considering the concordant expression of *SOX2* with its overlapping, It has been suggested that *SOX2OT* functions in *SOX2* regulation [18]. There are also some evidences supporting its function in regulation of the cell cycle in a polycomb-group protein, EZH2 dependent manner [17]. However, the underlying mechanism of *SOX2OT* function in cancer progression and differentiation appeals more investigations.

Preliminarily, we investigated two transcriptome resources to find out the most appropriate sample origin for SOX2OT functional analysis. According to the GENEVESTIGATOR software [19], SOX2OT gene expression is mostly reported to be de-regulated in brain and lung tumors (Additional file 1: Figure S1A). indeed, in a computationally reconstructed portrayal of human transcription database resource (MiTranscriptome) [20]; *SOX2OT* expression is reported to be mostly associated with the two cancer types of glioblastoma and lung carcinoma (Additional file 1: Figure S1B). Previously in our laboratory, we observed that SOX2OT inhibition can significantly decrease lung [21] and brain (un-published yet) cancer cell colony formation ability with a minor cell cycling disturbance. Then in this study, we aimed to explore the transcriptome changes in the SOX2OT knocked down glioblastoma and lung adenocarcinoma cell lines with the RNA sequencing to clear the cellular function of SOX2OT long non-coding RNA in cancer cells.

## Methods

### Cell culture

A549, human lung adenocarcinoma cancer cell line and U87-MG, human glioblastoma cell line were obtained from pasture institute (Tehran, Iran). Cells were cultured in RPMI 1640 (Invitrogen, Gaithersburg, MD) supplemented with 10% fetal bovine serum (Invitrogen, Gaithersburg, MD) and 100 IU penicillin-100 μg streptomycin per ml (Invitrogen, Gaithersburg, MD) in a 98% humidified 5% CO_2_ incubator. The cancer cells were used for transfection process with siRNA and gene expression analysis as following.

### RNA interference and transfection

Considering previous recorded high expression level of SOX2OT in cancer cells, RNA interference approach was used to investigate SOX2OT associated cellular functions. A previously reportedSOX2OTtargetingsiRNA (5′-GGAGAUUGUGACCUGGCUU-3′) [18] was synthesized. For the siRNA transfection, approximately 5 × 10^5^ cells were seeded at six well tissue culture plates. After 24 h, the cells (about 80% confluent) were transfected with control siRNA (Sanatacruze, s-c37007, 10 nM), or SOX2OT targeting siRNA (synthesized by Bioneer, 50 nM) using Lipofectamine 2000 according to manufacturer’s protocol. 48 h later, the cells were harvested and were stored in − 80 °C for subsequent gene expression analysis or RNA sequencing.

### RNA-sequencing

The total RNA of control and treated A459 and U87-MG cells were extracted by Trizol reagent (Invitrogen, Carlsbad, CA) according to manufacturer’s protocol. The RNA libraries were prepared after rRNA cleanup, according to the BGI standard pipeline. Sequencing of the four synthesized RNA libraries were carried out by Illumina Hiseq 2000 sequencing system with paired end sequencing method resulted to approximately 25,000,000 reads with 90 base pairs fragments in length. Primary sequences were checked for quality and then were aligned to the reference human genome (hg38) release using the bowtie2 [22] tool. To find out the differentially expressed genes (DEGs) between control-siRNA and SOX2OT-siRNA treated of each cell line separately, TopHat and Cufflinks pipeline was executed. The normalized abundance of the transcripts were reported as FPKM (Fragments Per Kilobase of transcript per Million mapped reads) and the differential gene expression was evaluated using Cuffdiff package (q-value < 0.05) [23].

### Functional enrichment of DEGs and network construction

The differently expressed gene lists (up-regulated or down-regulated) were used for gene ontology (GO) term enrichment by BINGO [24] software. For more confirmation, the common DEGs (n = 208 in both cell lines) were extracted and used for gene network analysis. The common DEGs were used for network construction using STRING protein interaction database [25] (with confidence score = 0.5) and then the resulted primary network (with 86 nodes) was again extended by GeneMANIA [26] according the co-localization, protein and genetic interaction, pathways and shared protein domains of the common DEGs resulting to more dense gene network (nodes = 196, 171 nodes in DEGs + 25 nodes added automatically). The edges in the final gene network was weighted based on biological process domain of GO and semantic similarity measure. The semantic similarity between genes was calculated based on rensik [27] method by using FastSemSim [28] tool (mixing strategy = max). Finally, the weighted gene network (weight ≥ 0.4) with 122 nodes was analyzed and visualized with Cytoscape 3.4 software.

### Gene expression measurement

To confirm the gene expression changes, seven genes enriched mainly in cell cycle regulation or mitotic progression were measured by more sensitive method such as qRT-PCR. Briefly, one microgram of total RNAs were DNaseI (Thermo Fisher Scientific, Inc) treated and were reverse transcribed using PrimeScript first strandcDNA synthesis kit (Takara) and random hexamer primers as described by the supplier. A volume of 2 μl of first strand cDNAs were used as template of real-time PCR.

The gene Expression analysis was carried out in Bioer (LineGene K) thermo cycler using qPCR Green Master with low ROX (Jena Bioscience, GmbH) and specific primers (Table 1). The cycling condition was as follow: enzyme activation at 95 °C (2 min), 40 cycles of denaturing at 95 °C (10 s), annealing at 60 °C (30 s) for *GNL3l* and *SOX2OT*; for the other genes; annealing at 58.4 °C (30 s), following extension at 72 °C (40 s). Final dissociation curve analysis was performed to ensure the specific PCR products.

**Table 1:** The primers sequence for q-PCR amplification

Gene symbol	Primer sequence
*SOX2OT* Gene ID: 347689	F: GGCTGGGAAGGACAGTTCG R: AGATGATCTTGCCAGGCGATC
*CDK2* Gene ID: 1017	F: CCCTTTCTTCCAGGATGTGA R: TCACCCCTGTATTCCCAGAG
*CDK2AP2* Gene ID: 10263	F: TGCCAGGCACTCTCTGACTA R: AGATCCGGCCTACCTATGCT
*GNL3L* Gene ID: 54552	F: TATCTTCTTGTGGCCCTTGG R: AGAGAGCAAGCAGATTTGAACC
*ACTR3* Gene ID: 10096	F: TTGAGTGGTGGTAGATTGAAGC R: CCAAACTGCATATCGCTGCAT
*SMC4* Gene ID: 10051	F: TGCAGAGGAATCCTTACCAG R: TGTTCAGCAATGTGACCATC
*INCENP* Gene ID: 3619	F: TCAGAACCAACTTTCTGGGG R: CAAGAAGACTGCCGAAGAGC
*GAPDH* Gene ID: 2597	F: GAGCGAGATCCCTCCAAAAT R: GGCTGTTGTCATACTTCTCATG

### Flowcytometry analysis

To investigate the cellular apoptosis and cell cycle progression, cells were harvested and then were stained with the annexin V/PI (Sigma-Aldrich) according the manufacturer’s recommendation for apoptosis evaluation; or were fixed in cold ethanol (70%) and then were stained with stain solution (PI 10 μg/ml) after treatment with triton X − 100 (0.1%) and RNAaseI (10 μl/ml, 30 min, 37 °C) for cell cycle assessment with the flowcytometry instrument (Partec GmbH, Münster, Germany).

### Statistics

The gene expression measurements were carried out in experimental replicates to decrease the artificial error. The SPSS v22 software was used to analyze data statistically. For statistical tests, 95% confidence interval and p value < 0.05 was considered. The one way ANOVAWAS used to compare means of measurements in treated cells to control. The mean ± SE has been presented in the graphs as error bars. For functional enrichment, the p value was corrected via false discovery rate (FDR) estimation.

## Results

### SOX2OT knock down leads to general gene expression de-regulation in both cancer cells

**Fig. 1:**
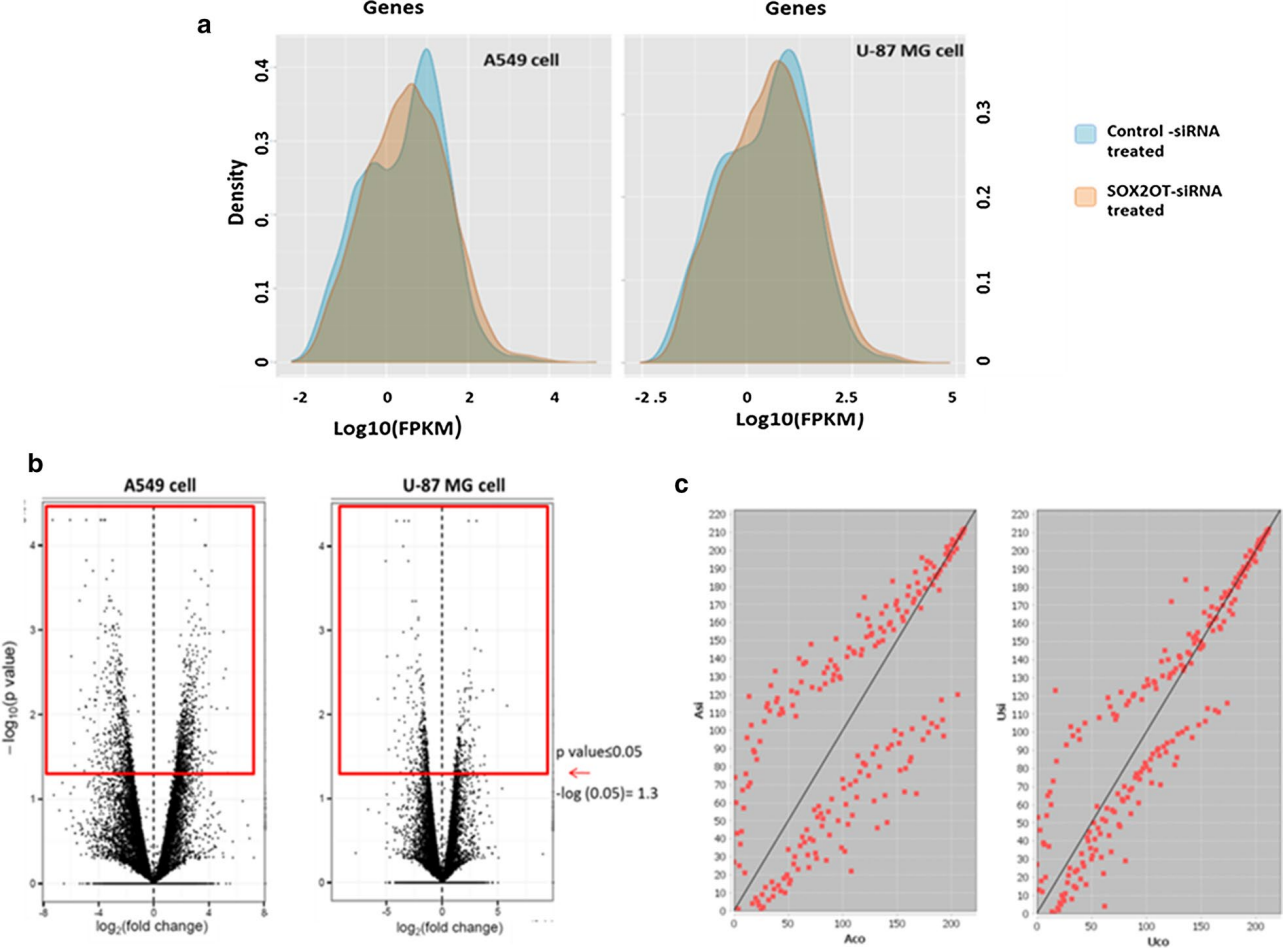
Differentially expressed genes in two cell lines (A549 and U87-MG) after SOX2OT knock down. The histogram shows the transcriptome of both cells are generally affected by the treatment (**a**). The volcano plot in the bottom describes the significant differentially expressed genes in each cell line (**b**) and the scatter plot for each cell line shows the dispersion of gene expression between control and SOX2OT knocked down cells (**c**). *FPKM* fragments per kilobase of transcript per million mapped reads

The RNA-sequences were analyzed as described above to investigate the differentially expressed genes for each cell line separately. More than 50,000 genes were mapped in transcriptome analysis, with 1588 genes significantly de-regulated in A549 (779 up and 809 down) and 609 genes significantly de-regulated in U87-MG (391 up and 218 down) after SOX2OT knockdown. As it is visualized by histogram and scatter plots in Fig. 1, SOX2OT knockdown generally changes the expression profile of each cancer cell affecting A549 lung cancer cell more than glioblastoma U87-MG cancer cell. However, regarding to the volcano plot, the fold change expression of some of DEGs is considered as significant.

The GO term enrichment of the significantly de-regulated genes in each cell line (Table 2) indicted that most of the enriched gene ontology terms are related to the cell cycle, DNA replication, and mitosis in A549 cell line (bolded in Table 2). More ever, in the U87-MG cell line, SOX2OT knockdown changes the expression of cellular genes related to neuronal differentiation, development and also cell cycle regulation and DNA replication (bolded in Table 2). These findings highlight the potential function of SOX2OT in cellular replication and mitosis process and cellular differentiation and development. Since the two sequenced cell lines are from different tissue origin with consequently different background transcriptome, it may describe the observed variances of DEGs between two samples.

**Table 2:** Gene ontology term enrichment of differentially expressed genes in the A549 and U87-MG SOX2OT knocked down cells

A549 cell line					U87-MG cell line				
GO-ID	p-value	Corr p-value	X	Description	GO-ID	p-value	Corr p-value	X	Description
**9987**	1.83E−05	9.26E−03	61	Cellular process	32502	4.68e−02	3.40e−01	96	**Developmental process**
**44085**	1.03E−04	1.06E−02	21	Cellular component biogenesis	6807	2.55e−03	3.19e−01	76	Nitrogen compound metabolic process
**7049**	6.16E−05	9.26E−03	17	**Cell cycle**	6950	2.55e−02	3.19e−01	58	Response to stress
**22402**	4.46E−04	1.90E−02	14	**Cell cycle process**	30154	4.98e−02	3.40e−01	53	**Cell differentiation**
**48519**	8.18E−05	9.85E−03	13	Negative regulation of biological process	42127	5.77e−03	3.19e−01	34	Regulation of cell proliferation
**43933**	2.12E−03	4.64E−02	13	Macromolecular complex subunit organization	6629	3.47e−02	3.40e−01	30	Lipid metabolic process
**278**	1.47E−04	1.18E−02	11	**Mitotic cell cycle**	6811	4.39e−02	3.40e−01	27	Ion transport
**51301**	5.24E−04	1.95E−02	11	**Cell division**	22008	2.10e−02	3.19e−01	25	**Neurogenesis**
**22403**	2.29E−03	4.73E−02	11	**Cell cycle phase**	48699	1.72e−02	3.19e−01	24	**Generation of neurons**
**10605**	3.55E−04	1.74E−02	10	Negative regulation of macromolecule metabolic process	8285	8.58e−03	3.19e−01	18	**Negative regulation of cell proliferation**
**45892**	6.13E−05	9.26E−03	9	Negative regulation of transcription, DNA-dependent	55086	2.43E−02	3.19E−01	14	**Nucleobase, nucleoside and nucleotide metabolic process**
**45934**	3.61E−04	1.74E−02	9	**Negative regulation of nucleic acid metabolic process**	6954	3.08e−02	3.40e−01	14	Inflammatory response
**51172**	3.61E−04	1.74E−02	9	Negative regulation of nitrogen compound metabolic process	7420	4.63e−02	3.40e−01	13	**Brain development**
**6260**	5.07E−04	1.95E−02	7	**DNA replication**	6916	3.19E−02	3.40E−01	10	Anti-apoptosis
**9628**	1.19E−03	3.18E−02	7	Response to abiotic stimulus	6260	4.82e−02	3.40e−01	9	**DNA replication**
**6261**	2.03E−03	4.59E−02	5	**DNA-dependent DNA replication**	1525	4.03E−02	3.40E−01	8	Angiogenesis
**10688**	2.93E−04	1.74E−02	2	Negative regulation of ribosomal protein gene transcription	51325	1.84e−02	3.19e−01	7	**Interphase**
**30837**	2.00E−03	4.59E−02	2	**Negative regulation of actin filament polymerization**	7265	2.41e−02	3.19e−01	7	Ras protein signal transduction
**32272**	2.00E−03	4.59E−02	2	Negative regulation of protein polymerization	82	4.27e−02	3.40e−01	4	**G1/s transition of mitotic cell cycle**
**8154**	2.00E−03	4.59E−02	2	**Actin polymerization or depolymerization**	48048	8.49e−03	3.19e−01	3	**Embryonic eye morphogenesis**

X: the number of genes in enriched GO term, corr p-value: FDR corrected p-value

To investigate the DEGs function, the top 400 genes of the expression signature profile of DEGs in U-87 MG cell line with p-value ≤ 0.02 was used as a template signature to find out the most similar signatures available in the public data bases using the GENEVESTIGATOR software (by Euclidean distance score). Interestingly we found that the log2 expression of the DEGs in U87-MG cell line after SOX2OT knock down is mostly similar to studies with Asthma perturbation (relative similarity score = 1.6), hypoxia (relative similarity score = 1.4) and exposure to cell cycle inhibitor chemicals like mitomycin and colchicine (relative similarity score > 1.3) (Additional file 1: Figure S2).

Furthermore, DEGs signature in the A549 cell after SOX2OT knock down mostly resembles conditions like: human ovarian tumors (relative similarity score = 1.28), smoking (relative similarity = 1), asthma (relative similarity > 1), hypoxia (relative similarity score = 1.15) and exposure to the anti-cancer drugs like: R549, (CDK inhibitor), echinomycin (HIF inhibitor) and zalypsis (double strand break inducer) (relative similarity score > 1.1) (Additional file 1: Figure S3). Studying the most resembling perturbation signatures can provide more clues to find out the importance of *SOX2OT* gene function in human diseases.

### Common genes de-regulated in both SOX2OT knocked down cancer cell lines

**Fig. 2:**
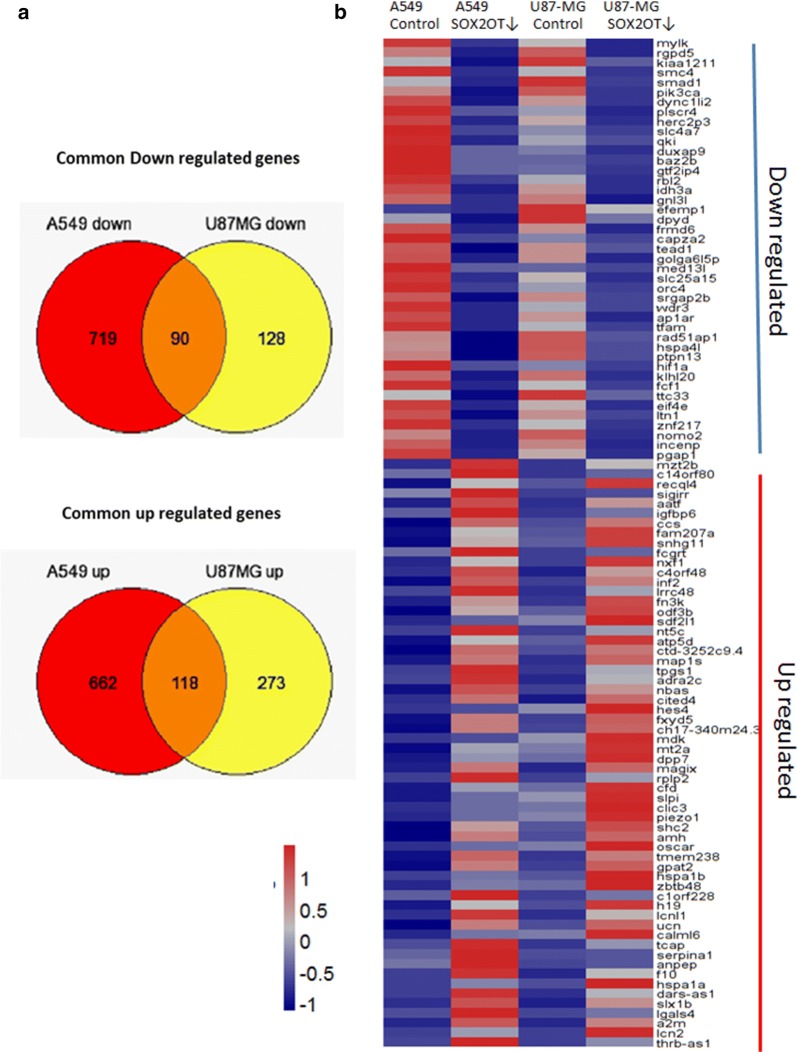
Common differentially expressed genes in both cell lines. The venn diagram presentation of DEGs was used to find out the gene list differentially expressed in both Lung adenocarcinoma, A549 and glioblastoma, U-87 MG cell lines (http://genevenn.sourceforge.net) (**a**). The heat map presentation of top common DEGs in siRNA treated or control cell line, which red: high expression value, blue: low expression value and white: mean level of expression value (**b**)

For more precision, we searched the differentially expressed genes in each cell line to find out the sharing gene list of DEGs in both target cell lines, and we named it: common DEGs. We found 208 genes differentially expressed in both SOX2OT knocked down cell lines. The Venn diagram represented that 90 genes are down regulated and 118 genes are up regulated upon SOX2OT inhibition in Lung cancer cell similar to glioblastoma cell (Fig. 2a). The gene expression value of each sample was used for heat map visualization using geWorkbench platform [29] (Additional file 1: Figure S4). The top differentially expressed genes ordered according the mean of fold change in both cells are shown in Fig. 2b. The GO enrichment of the common DEGs was done with both Bingo and GeneCodis [30] with default parameters and it is available in Additional file 1: Table S2.

The resulted common DEGs list was used for further enrichment and biological process annotation. The most relevant gene ontology terms to Nucleotide binding or DNA replication and cell cycle was summarized in Table 3.

**Table 3:** The cell cycle related gene ontology enriched genes in the common DEGs

GO term	p value	Enriched genes
Cell cycle (KEGG)	0.004	RBL2, ORC4, **CDK2**, MAD1L1
Nucleotide binding (MF)	0.0001	R3HCC1, HRAS, CARS2, NXF1, ORC4, MVD, G3BP2, PIK3CA, HIPK1, HSPA1B, RHOT1, **ACTR3**, RAP1B, DDX21, **GNL3L**, **CDK2**, MYLK, MRPL23, HSPA4L, CDC34, CHUK, PMVK, PRKD2, **SMC4**, HSPA1A, DYNC1LI2
G2/M transition of mitotic cell cycle (BP)	3.23E−06	TAF2, **CDK2**, NEDD1
Mitotic cell cycle checkpoint (BP)	0.0018	ORC4, **CDK2**, MAD1L1
G1/S transition of mitotic cell cycle (BP)	0.008	ORC4, **CDK2**, CDC34, EIF4E
Mitotic cell cycle (BP)	0.026	ORC4, **CDK2**, MAD1L1, **INCENP**, NEDD1

The most related pathway to SOX2OT inhibition was renal cell carcinoma (Kegg: 05211, corrected p value = 0.004953) with 5 genes differentially expressed in RNA sequences of both cells (*hif1a*, *hras*, *pik3ca*, *rap1b*, *vhl*). However, the cell cycle pathway (Kegg: 04110, corrected p value = 0.03) including the proliferation associated genes (*rbl2*, *orc4*, *cdk2*, *mad1l1)*, acquired less score.

**Fig. 3:**
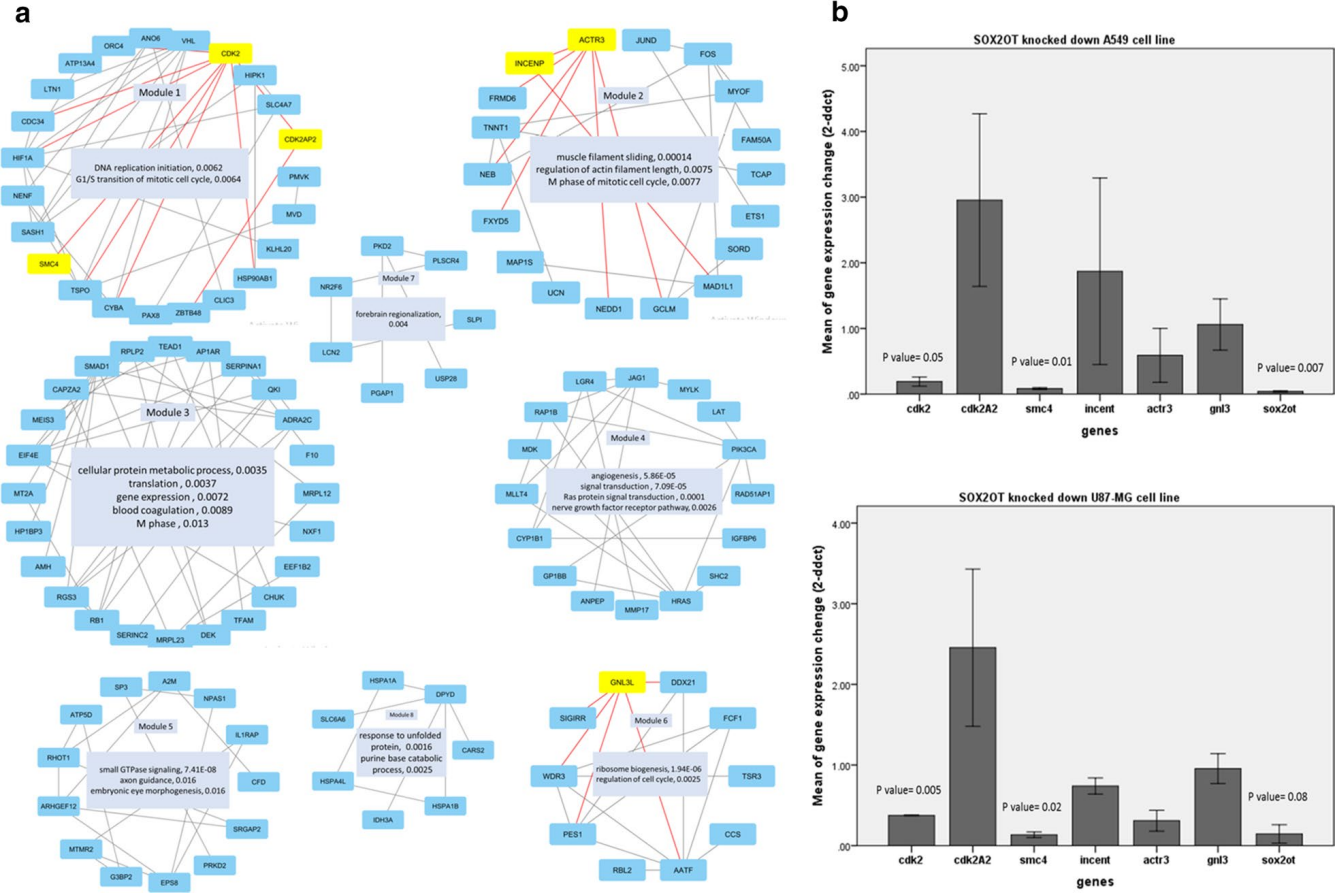
Gene network analysis in SOX2OT knocked down cells. **a** The graphic presentation of modules in the SOX2OT knocked down differentially expressed gene network. For ease of annotation, the network was first clustered with GLay and then the modules were used for biological process enrichment with GeneCodis. The most significant enriched GO terms are labeled with FDR corrected hyper geometric p value. The selected nodes for following expression confirmation are highlighted in the modules. Notice their edges in red color. **b** The gene expression fold changes confirmed with QRT-PCR in SOX2OT knocked down cells. The bars represent mean ± SE

The gene network of common DEGs was constructed according the method described in “RNA-sequencing” section. The network was then clustered with the GLayalgorithm [31] to find the most important modules in the network. For more confidence, the edges with weight lower than 0.4 were removed from the network resulting to filtered network with 122 nodes. The clustering resulted to the number of eight modules with the most relevant genes correlated, and the two modules with less than 3 nodes were ignored. Then each module was annotated for functional enrichment and the resulted GO terms were used to describe each module (Fig. 3a). Interestingly, we found four gene clusters (modules: 1, 2, 3 and 6) associated with cell cycle or DNA replication and two related with brain or eye development (modules: 5 and 7). The network was then analyzed to find the most important genes, according to the weighted betweenness centrality measurement [32]. Then the 10 top sorted genes with the highest betweenness were extracted in Table 4. According to the modules 1, 2 and 6 (with cell cycle related genes p value < 2 × E − 2) and the top 10 hub genes in the network, we selected the number of six genes (bolded in Table 3) for qRT-PCR validation.

**Table 4:** The 10 hub genes with highest betweenness

No.	Symbol	Name	Betweenness	Degree
1	**CDK2**	Cyclin-dependent kinase 2	2892.27	19
2	TSPO	Translocator protein	1921.052	16
3	EIF4E	Eukaryotic translation initiation factor 4E	1787.614	12
4	SERPINA1	Serpin peptidase inhibitor, clade A	1535.751	12
5	HSPA1A	Heat shock protein family a (hsp70) member 1a	1353.741	6
6	SMAD1	Smad family member 1	1157.476	12
7	**ACTR3**	ARP3 actin-related protein 3 homolog	1072.58	10
8	HIF1A	Hypoxia-inducible factor 1-alpha	980.6956	12
9	HRAS	Harvey rat sarcoma viral oncogene homolog	965.7061	12
10	**GNL3L**	G Protein Nucleolar 3 Like	945.4339	8

### Cell cycle associated genes are de-regulated in both cell lines

Previously, we reported that*SOX2OT* knockdown can limit A549 lung cancer cell proliferation with minimal cell cycle perturbation. Here we first measured the effect of SOX2OT inhibition in U-87 MG glioblastoma cancer cell apoptosis and cell cycle progression. Same as our previous report in A549 [21], we observed no apoptosis in SOX2OT knocked down U-87 MG cell line (Additional file 1: Figure 5A), however the population of G2/M cells were increased approximately 5% in SOX2OT siRNA treated cells (Additional file 1: Figure 5B). According to the previous reports of SOX2OT function in cell cycle regulation [17, 21], we decided to focus on the DEGs related to cell cycle regulation. The qRT-PCR results confirmed the down-regulation of the mitotic cell cycle regulators such as *CDK2* and *SMC4* in both lung and glioblastoma SOX2OT knocked down cancer cell lines, while the *CDK2AP2* which is also known to interact and inhibit *CDK2* [33], is up-regulated more than two times in treated cells (Fig. 3b). The gene expression changes of other targeted genes (inner centromere protein, guanine nucleotide binding protein-like 3 and actin-related protein 3) were not significant; however we observed the increased GNL3L expression and decrease ACTR3 expression in both SOX2OT knocked down cancer cell lines same as RNA sequencing results. The *GNL3L*, which promotes the mitotic Telomeric repeat binding factor 1 [34], and *ACTR3*is constituent of the ARP2/3 complex of actin nucleator [35] are both important in mitotic progression.

## Discussion

Recently another cancer associated lncRNA, *SOX2OT*, has been discovered as an embryogenesis and central nervous system regulator [14, 36]. SOX2OT is over expressed in human cancer tissues of lung, esophagus and breast when comparing to normal and it’s over expression in tumors is associated with *SOX2*, which is located within SOX2OT [16–18]. We found that according to the KEGG enrichment of the common DEGs deregulated upon SOX2OT inhibition; the pathways related to cancer (Kegg: 05200) is one of the most significant enriched pathway (p-value = 0.009285) with 6 genes (*HIF1A*, *HRAS*, *CHUK*, *PIK3CA*, *CDK2*, *VHL*). This finding highlights *SOX2OT* potential function in cancer development and may help to explain how SOX2OT up-regulation can affect tumorigenesis.

It has been reported that the expression of both SOX2 and its overlapping transcript are dynamically co regulated in embryogenesis [14] and decline during stem cell differentiation [18]. Exogenous expression of *SOX2OT* increase *SOX2* more than 20 fold [16]. Furthermore, *SOX2OT* knockdown is concordant with *SOX2* decline in cancer cells [17, 18, 21]. The aforementioned evidences describe why it has been proposed that *SOX2OT* regulates *SOX2*; however there is no evidence supporting the direct interaction of these two transcripts.

Notably, it has been reported that 3q27 genetic mutation (SOX2 and SOX2OT containing region) is associated with congenital CNS and eye development disorders [36]; and the normal human brain and lens tissues have been recorded to posse the highest SOX2OT expression level. In vertebrate embryos, SOX2OT expression is dynamically regulated highly expressed in developing central nervous system [14]. Concordantly we found some central nervous system development and maintenance key regulators including: *JAG1* [37], *MDK* [38], *PKD2* [39], *HES4* [40] and the eye formation regulator such as *HIPK1* [41], de-regulated along with SOX2OT inhibition. These findings may suggest a mechanism for SOX2OT in CNS and eye development.

It has been reported that *SOX2OT* knockdown leads to a G2/M arrest and proliferation inhibition in HCC827 and SKMES-1 lung cancer cell lines with a *EZH2* poly comb protein dependent cyclinB1 and cdc2 regulation mechanism [17]. In our previous study, we found similar *SOX2OT* knockdown derived anti-proliferative and cell cycle effect in and A549 cells [21] and here in U-87 MG cells. Our results illustrated that gene networks of cell cycle progression or cell proliferation (including: *CDK2* and *SMC4*) are affected in both SOX2OT knock downed cancer cells. However, we did not observed cyclinB1 or EZH2 among SOX2OT associated common DEGs that might be related to low precision of the high throughput RNA-sequencing method. Indeed, we observed other cyclin dependent cell cycle regulators including: cdc6, cdc27, ccnt2, ccnt1 and ccng2 downregulated and cdc37 upregulated only in A549 cell line transcriptome but not in U87 MG (which is not shown). The functional enrichment of transcription factors or gene regulators revealed that, SOX2OT knock down can potentially change cellular expression of transcription factors which is listed in Additional file 1: Table 3. One of the key cell cycle regulator transcription factors, rbl2 is included among the SOX2OT associated transcription factors; however, it needs more investigation to find out their interaction.

**Fig. 4:**
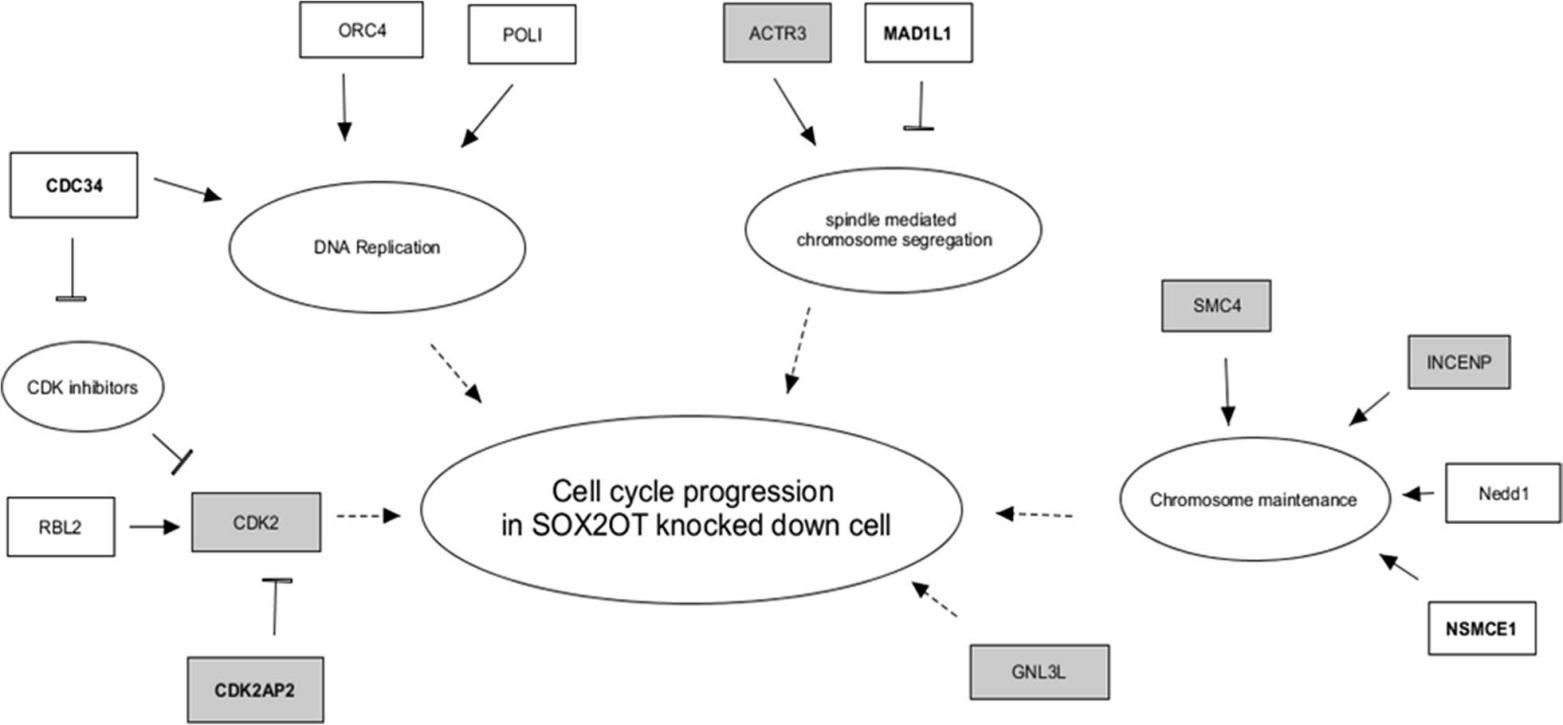
The proposed cell cycle gene network changes associated with SOX2OT adopted from common DEGs. The up-regulated genes are presented with bold font and the qRT-PCR checked genes are showed in gray boxes which only the expression decrease of SMC4, CDK2 and SOX2OT was significant

The predicted schematic presentation of the interaction between cell cycle associated common DEGs is summarized in Fig. 4. The interaction between candidate transcription factors and the confirmed DEGs suggest that RB1 and RBL2 are two predicted main transcription factors associated with *SMC4*, *CDK2* and *INCENP*, *GNL3L* (indirectly) expression regulation in SOX2OT knocked down cells.

It has been reported that SOX2OT over-expression in breast cancer cell line can restore the G2/M arrested paclitaxel treated cancer cells [16]. SOX2OT is also associated with the hepatic cancer cell migration and metastasis [42]. Our findings suggest some actin cytoskeleton organization (*EPS8*, *INF2*, *CAPZA2*, *TCAP*, *PLEK2*, *HRAS*, *ACTR3*) or tubule counterparts (*MAP1S*, *MTMR2*) which provides more evidence to support the probable SOX2OT function in cytoskeleton regulation.

Indeed SOX2OT is reported to bind to PIN2/TERF1-interacting telomerase inhibitor 1 (PINX1), in vitro [43]; which can stabilize Telomeric repeat binding factor 1 (TRF1) [44]. GNL3L that is down regulated in SOX2OT inhibited cell can also bind and stabilize TRF1 protein mediatingits mitotic increase and mitosis progression [45]. Also we did not found TRF1 or PINX1 in DEGs since our study was performed at transcription level, then for more completed SOX2OT functional analysis; proteomics or cellular component structure investigation is suggested.

## Conclusion

Altogether, our results illustrate that *SOX2OT* knock down can significantly alter the gene expression profile of cancer cell lines targeting the cell cycle, proliferation and cytoskeleton related genes. This novel potential function of *SOX2OT* promises new insights in cancer therapies; however more investigation is necessary to clear the underlying mechanism of *SOX2OT* relevance to mitotic cell cycle regulation or embryonic development.

## Additional file

**Additional file 1: Figure S1.** The most associated cancers with the SOX2OT long non-coding RNA. The GENEVESTIGATOR tool was used to find out the most related conditions reported in databases to be associated with SOX2OT expression changes. The top significant conditions are listed in left including two types of brain and lung cancers (A). The schematic presentation of the SOX2OT expression level in different tissues adopted from the MiTranscriptome (B). **Figure S2.** The most similar conditions to the DEGs in SOX2OT knocked down U87-MG cell line (log2 expression). **Figure S3.** The most similar conditions to the DEGs in SOX2OT knocked down A549 cell line (log2 expression). **Figure S4.** The heat map presentation of all the significant common DEGs in siRNA treated or control cell line, which red: high expression value, blue: low expression value and white: mean level of expression value. **Figure S5.** SOX2OT inhibition effect in apoptosis and cell cycle of cancer cell lines. (A) The apoptotic response of the SOX2OTknocked down U-87 MG was compared with control cells with annexin V/PI staining. As is shown, no annexinV-positive cells (FL1) were detected. (B) The flow cytometry evaluation of PI-stained (FL3) cell cycle progression in U-87 MG cell is illustrated in tables for control and SOX2OT knocked down cells. **Table S1.** The complete common DEGs (P value ≤ 0.05) in both cancer cell lines (A549 and U-87MG). **Table S2.** Functional gene enrichment results of the common DEGS carried out by Bingo or GeneCodis. **Table S3.** Functional enrichment of transcription associated genes.
